# Embodiment and Its Influence on Informational Costs of Decision Density—Atomic Actions vs. Scripted Sequences

**DOI:** 10.3389/frobt.2021.535158

**Published:** 2021-04-12

**Authors:** Bente Riegler, Daniel Polani, Volker Steuber

**Affiliations:** ^1^ Sepia Lab, Adaptive Systems Group, School of Engineering and Computer Science, University of Hertfordshire, Hatfield, United Kingdom; ^2^ Biocomputation Research Group, School of Engineering and Computer Science, University of Hertfordshire, Hatfield, United Kingdom

**Keywords:** agent control, information theory, embodiment, Reinforcement learning, morphological computing

## Abstract

The importance of embodiment for effective robot performance has been postulated for a long time. Despite this, only relatively recently concrete quantitative models were put forward to characterize the advantages provided by a well-chosen embodiment. We here use one of these models, based on the concept of relevant information, to identify in a minimalistic scenario how and when embodiment affects the decision density. Concretely, we study how embodiment affects information costs when, instead of atomic actions, scripts are introduced, that is, predefined action sequences. Their inclusion can be treated as a straightforward extension of the basic action space. We will demonstrate the effect on informational decision cost of utilizing scripts vs. basic actions using a simple navigation task. Importantly, we will also employ a world with “mislabeled” actions, which we will call a “twisted” world. This is a model which had been used in an earlier study of the influence of embodiment on decision costs. It will turn out that twisted scenarios, as opposed to well-labeled (“embodied”) ones, are significantly more costly in terms of relevant information. This cost is further worsened when the agent is forced to lower the decision density by employing scripts (once a script is triggered, no decisions are taken until the script has run to its end). This adds to our understanding why well-embodied (interpreted in our model as well-labeled) agents should be preferable, in a quantifiable, objective sense.

## 1 Introduction

Research in robotics, while dominated by kinematic and dynamic control laws, has increasingly identified the importance of embodiment for effective operation (Brooks, 1991; Paul, 2006; Paul et al., 2006; Pfeifer et al., 2007). Despite being closely related to the old historical research into cybernetics for robotic control, the study of embodiment still ekes out a relatively marginal existence compared to the mainstream techniques. Despite the obvious appeal of the postulate that well-constructed embodiment considerably simplifies robotic tasks and impressive examples (McGeer, 1990; Collins et al., 2001; Pfeifer et al., 2007; Beer, 2014), there has been no established overall agreed-upon formal framework with which to characterize and quantify embodiment and to demonstrate the relevance of this concept for robot design, expressed in objective metrics allowing to compare embodiment designs with each other. Here, we use information-theoretic measures as cognitive cost to compare embodiments. In particular, we study the effect of the encoding of actions and multistep action scripts (Riegler and Polani, 2019) using the “twisted world” method (Polani, 2011) on the informational cost of the agent.

### 1.1 Informationally Cheap Stimulus Responses Through Embodiment

Much of the historical discussion and motivation for studying good embodiment comes from studying biological examples (Varela et al., 2016). Living animals interact with their environment regularly, be it to find food or to escape their predators. In order to act effectively, the animal needs to sense its environment and take decisions based on the stimuli. Both require neural activity which is energetically expensive. In contrast to traditional AI/robotic systems, finding a compromise between saving deliberative resources and optimizing some explicit value function becomes an essential component of any biologically plausible survival strategy (Laughlin, 2001; Polani, 2009). However, given that the neural system of animals is one of the main consumers of energy (Vergara et al., 2019) (for a human, about 20% is used by the brain alone while it only contributes about 2% of the body weight), this implies that maximizing the efficiency of information processing is a major route to minimize energy consumption, argued in previous studies (Klyubin et al., 2004; Genewein et al., 2015). Thus, in biologically plausible decision modeling, the question of how to minimize deliberative cost becomes paramount. In particular, each reduction of the amount the agent needs to sense and to make decisions is an advantage for the organism.

In recent years, several attempts have been made to formally incorporate and measure the impact of embodiment on information processing in artificial agents. In (Montúfar et al., 2015), the authors show how controller complexity interacts with physical and behavioral constraints and that controller complexity can be reduced if the latter is suitably adapted, meaning well-embodied. Another study defines embodiment as how actions that have an effect on the world are encoded or labeled in the internal representation of the controller (Polani, 2011) while keeping the agent’s external situatedness the same. Under the assumption that the agent has limited information-processing resources, it shows that this internal labeling can drastically affect the performance of an agent, even if the actual dynamics of the world is completely unchanged. Or, vice versa, to achieve the same performance level, the required information processing resources can drastically depend on the embodiment in the sense of this model. Both papers, despite their differences, approach the question of the quality of an embodiment from the philosophy that any type of decision-making takes not only the pure quality of the decision into account but also the informational cost required to achieve it. The central message is: A good embodiment is one that makes decisions informationally cheaper, less complex, and thus more robust. Hence, a good embodiment reduces the agent’s (in our case: informational) effort to take decisions. Throughout this paper, we will therefore always take the information cost of decisions into account.

### 1.2 Limited Amount of Stimulus Responses Through Embodiment

In natural organisms, we find two other special mechanisms to save information processing costs: *reflexes* and *fixed action patterns* (Dewsbury, 1989). Cascades of multiple actions triggered by only one signal reduce the decision density, as the behavior, once started, progresses without requiring decisions based on intermediate stimuli.

Reflexes are complex reactions which are triggered by simple (often peripheral) neural control without the involvement of higher brain areas (Hurteau et al., 2018). Many reflexes involve just one body part, such as the eye-blink-reflex; others like the withdrawal reflex coordinate multiple body parts (Willows and Hoyle, 1969). These actions produce a potentially complex behavior with very limited information processing and fast processing speed due to directly linked neurons (Hurteau et al., 2018).

Similarly, fixed action patterns require little or no sensory input and are found often in biological organisms. Such behaviors comprise complex movements across a whole species that vary only in intensity (decided at the start of the movement) but not in form (Páez-Rondón et al., 2018). One example is the final jump of a cat to its prey (Schleidt, 1974; Dewsbury, 1989): The prey’s (future) position is set as the target position when beginning to jump and the cat will miss the prey if the latter moves surprisingly. Variations exist across individuals but these are fairly small. Importantly, fixed action patterns run until completion once started, suggesting they are run by “hard-wired” neural controllers. This indicates that regularly executed actions and action sequences can therefore profit from automation or “scripting” in organisms—being condensed into a “ritualized” sequence that does not respond to any feedback from the environment once triggered. Both, reflexes and fixed action patterns are often specific skills, required for a particular species or the current “life circumstances” of an individual.

### 1.3 Embodiment Considering Both Strategies to Save Information Processing

Under the assumption that organisms attempt to minimize information processing (Laughlin, 2001; Klyubin et al., 2004; Vergara et al., 2019), fixed action patterns would offer a natural route to reduce the decision density and overall information processing cost. We hypothesize significant savings on informational cost, especially when combined with embodiments minimizing the information processing per single stimulus-response (decision). In the present paper, we will develop a minimalistic quantitative model to study the essential links between information parsimony, embodiment, and artificial fixed action patterns.

Concretely, we approach action sequences by combining already existing (basic) actions into larger “super”-actions that do not require sensor input while running and are thus similar to the behaviors of fixed action patterns. These are what we call “scripts”. We then investigate the efficacy of these action scripts using the information-theoretic framework from (Polani et al., 2006; Polani, 2011) and investigate in which way a “well-chosen” agent-environment interaction structure, (i.e. embodiment in the sense presented above) impacts the cognitive cost of actions and scripts. We will see in the results that using scripts makes decisions more costly in some cases but does not in others. Furthermore, the results show that the embodiment of basic capabilities has a large impact on the cognitive cost to employ those scripts in any of our scenarios.

This work is organized as follows. Section 2 defines the nomenclature and basic concepts we will revisit throughout the paper. In Section 3 we define scripts, twisted worlds, and the model for costing decisions formally. Section 4 presents the methodology used to evaluate the models. In Section 5 we present the result which will be discussed in Section 6 and end with the conclusion in Section 7.

## 2 Nomenclature

### 2.1 Probabilistic Quantities

We use uppercase letters for random variables *A*, *S*, lower case letters to define a concrete value *a*, *s* and s, A for their respective domain. In this paper all domains are finite. The probability for a random variable *S* to assume a value s∈s is denoted as P(S=s) or directly p(s) when there is no ambiguity. When two different random variables have the same domain such as s, we will define the random variables *S* and $\tilde{S}$ or p(s) and $\tilde{p}\left(s\right)$ for their distributions to distinguish them. We will write $S_{t}$ for a random variable at time *t* (and $s_{t}$ for its value). Its successor state will be denoted as $s_{t+1}$. Sometimes we will use the common shorthand version *s* and $s^{\prime }$ in its place, especially when the particular time *t* is not of importance.

### 2.2 Markov Decision Process

We use Markov Decision Processes (MDPs) to model the perception-action cycle assuming that the world state can be—in principle—fully known.

For the definition of an MDP we follow (Sutton and Barto, 2018). Given a state set s and for every state s∈s an action set A(s), an MDP is given by a tuple $\left(P_{s,a}^{s^{\prime }},R_{s,a}^{s^{\prime }}\right)$ which for all $s,s^{\prime }\in s$ and all a∈A(s) defines a probability of performing action *a* in state *s* and ending in state $s^{\prime }$ in $P_{s,a}^{s^{\prime }}$. $R_{s,a}^{s^{\prime }}$ defines a reward function, mapping a certain reward to the agent when this particular transition occurs. Note that the reward will in general depend on the resulting state $s^{\prime }$ and not only on the initial state *s* and action *a* chosen.

A policy is denoted as follows π(a|s), the action *a* to choose in the state *s*. Following the behavior specified by the policy, the agent will accumulate a reward depending on the state $s_{t}$. For $\pi \left(a|s_{t}\right)$ the resulting reward is dependent upon the tuple ($s_{t}$, $a_{t}$, $s_{t+1}$) and given by $r_{t}$. Following the policy to a goal state (an absorbing state where no further reward is accumulated, i.e., effectively a run stops there) will accumulate a certain return $G_{t}=\sum_{t}^{\infty }r_{t}$.

We will assume episodic tasks throughout the paper. Therefore, we do not need to include a discount factor in the cumulated reward (we ignore possible pathological “runaway” phenomena, and limit our experiments to finite worlds). We see the policy that maximizes the future expected return (the value function $V^{\pi }\left(s\right)$) for whichever state s∈s the agent is in give policy π.

We further will write $V_{g}\left(s\right)$ to be the value function induced when g is specified as goal state (or set of goal states G). We assume a negative transition reward, (i.e. a cost) for each transition, except once the goal is reached, when the agent stops and the episode ends. $V_{g}\left(s\right)$ which is given by the optimal value from state *s* to state *g* with respect to the reward function and measures a type of (negative) distance from s to g (or G).

The *Q*-function Q(s,a) permits this to be expressed as a value assigned to a state/action pair instead of only a state:

$$Q^{\pi }\left(s,a\right)=\sum_{s^{\prime }\in S}P_{s,a}^{s^{\prime }}\cdot \left[R_{s,a}^{s^{\prime }}+V^{\pi }\left(s^{\prime }\right)\right].$$ (1)

From Q(s,a) one obtains directly the policy π(a|s). Throughout the paper we use only the optimal value V(s) to calculate $Q^{*}\left(s,a\right)$, and thus the resulting policy is the optimal policy and thus the solution of the MDP.

### 2.3 Shannon Information and Mutual Information

Shannon’s information theory describes the potential information content of a random variable using its entropy which measures the uncertainty about the outcome before the actual value has been observed. Throughout the paper we will use extensively use the concept of the *mutual information* measuring how much information *Y* provides about *X* (and vice versa) defined as:

$$I\left(X;Y\right)=D_{KL}\left(P_{\left(X,Y\right)}∥P_{X}⊗P_{Y}\right),$$ (2)

where $D_{\text{KL}}$ is the Kullback–Leibler divergence of *X* and *Y*. This quantity is non-negative and vanishes if and only if *X* and *Y* are independent, that is when *X* does not provide any information about *Y* and vice versa. For further details see (Cover and Thomas, 1991).

## 3 Models

### 3.1 Scripts

Based on the basic action $\left(A_{b}\right)$ of an agent, *scripts* are an extension of the action space of the MDP framework, a construct similar to fixed action patterns as found in biology. As such, Scripts are completely independent of the state space of the MDP. Once created, we assume the scripts to be available in every state as new actions available to the agent. Scripts do not create new capabilities for the agent since they are completely built upon the existing basic actions.

Scripts combine multiple actions originally taking one time step into one new action. From the agent’s point of view, the time steps inside the scripts are not accessible. Thus, scripts provide a temporal abstraction similar to options (Sutton et al., 1999) and sub-goals (van Dijk and Polani, 2011). Unlike scripts, these approaches are also dependent on the state space as the ending condition of an option and sub-goal are linked to specific states of the MDP.

#### 3.1.1 Definition of Scripts

Agents in reinforcement learning are assumed to have some basic (atomic) actions. These actions vary depending on the task and the granularity of the model (Sutton and Barto, 2018). Here we adopt this assumption and call the set of basic actions $A_{b}$.

We define scripts as a sequence of n>1 basic actions with strictly more than one action:

$$\text{Scripts}=\left\{{\left(A_{b}\right)}^{n}\right\}.$$ (3)

This defines a single script as an ordered tuple, or better, a sequence of all its components, for example $\text{script}=\left(a_{0},a_{1},a_{2},\ldots ,a_{n-1}\right)$. We do not consider the basic actions on their own to be scripts. A script has to have a finite length. Scripts do not introduce interaction modalities that were not available before but instead, the agent gets the ability to decide on the compound behavior at a single point in time, namely the time when the script is triggered. This is especially suitable to carry out basic actions which always follow each other in a fixed sequence such as the dive of a falcon (Schleidt, 1974).

The new actions space in our model contains all basic actions and all scripts up to a certain length: $A_{b}\cup {\left(A_{b}\right)}^{1}\cup {\left(A_{b}\right)}^{2}\cup \ldots \cup {\left(A_{b}\right)}^{n}$. In our model, there is no mechanism to add or remove a script to or from the action space. Of course, the algorithm may decide to effectively remove a script or action from use by setting its selection probability to zero.

#### 3.1.2 New Perception-Action Loop

**FIGURE 1:**
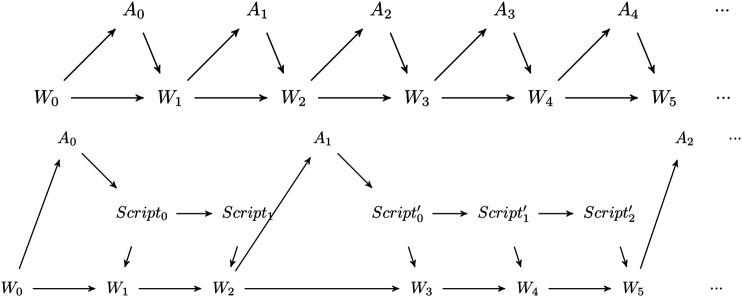
Top: Bayes Network of the perception-action loop of an agent with only basic actions. During every time step, the agent perceives the world. Bottom: The Bayes Network of the agent’s perception-action loop with an intermediate script-layer. The agent is only able to perceive and react to the world at some points in time after the script is finished.

The scripts now modify the Bayes Network of the perception-action loop. As in a perception-action loop with no scripts available, the state of the world changes every time step, depending on its previous state and the basic action performed by the agent (see Figure 1, top). The agent, though performing a basic action every time step, only takes decisions at some time steps and is only perceiving the world at these defined times as the script runs fully in open-loop fashion (Distefano et al., 2012), a major difference to options (Sutton et al., 1999). The possibility to augment this with conditions to interrupt a script is not studied here. The scripts create a new layer in the perception-action loop allowing one to sequentially perform actions impacting the world but without taking into account any perception by the agent (see Figure 1, bottom).

With this, the intermediate layer in the form of scripts, this layer now takes care of the sequence of actions and the timing of when to perform these. We emphatically shall not consider the processing and management cost required for running the script itself once it started and assume the necessary hardware (such as in the form of a suitable control circuit) is part of the agent’s given design.

#### 3.1.3 Hidden Temporal Abstraction

With the basic actions unchanged, scripts containing more basic actions take more time steps to complete than shorter scripts or basic actions. Thus, scripts provide a form of temporal abstraction.

As a consequence, this forms a semi-MDP as the time steps of the world no longer match the time steps (actually decision steps) of the agent. For simplicity reasons, we will not model the length of the scripts in time. Instead, we will only model the time steps when the decision of the agent occurs and hide the difference in “world-time-steps” in the reward (in our case, cost) function: A script simply gives as much reward as if all basic actions it contains were executed in sequence, implying it took longer. From the agent’s perspective, this is just a different cost but with one time step. Effectively, the agent is only aware of those time steps in which it has to decide, even though the world itself has finer time steps. Hence, by only modeling the time steps of the agent, we can treat it as an MDP instead of a semi-MDP.

#### 3.1.4 Effects on Value Optimization

Considering the scripts plainly as extensions of the action space, they all follow the definition of an action in the traditional MDP-framework with all state transitions being defined as $\left(s^{\prime },r|a,s\right)$. For every pair of state and (super-)action the agents moves to a state and gets a reward as defined in the previous section. This permits us to use value iteration in the precisely same way with or without scripts, by either including or excluding the scripts with length n>1 in the set of actions.

With scripts available, every path can either be achieved by a script moving along the full path or shorter scripts and basic actions each only moving along part of the path. This is also true for every optimal path. Hence, the resulting value-optimal policies exhibit multiple optimal actions (basic action or scripts) in every state and could be probabilistically selected even though they might use the same basic action, whether on its own or inside a script.

The new action space is also significantly bigger. In fact, the potential action space can grow exponentially with the maximum script length permitted. Hence, the computation time will thus grow as well. This renders the optimization over the whole action space that would contain every possible script infeasible for longer script lengths. This also makes identifying ‘good’ and ‘bad’ scripts and preselecting the relevant actions and scripts in the action space accordingly an important topic for the future.

### 3.2 Twisted World

The state space of an MDP does not have an initial interpretation as anything else than a set of discrete, a priori unrelated states. Linking these states using labeled actions often introduces additional semantics in the labels of the states and actions. In typical scenarios, e.g., in navigation, the same action label always leads to the “same” (or an analogous) change of the state. This injects a semantics into the labeling which is not part of the core MDP formalism. The goal of the twisted world, defined by (Polani, 2011), and subject of this section, is to investigate the bias of a particular internal labeling, and thus any embodiments.

In traditional MDP models as previously defined, any state could have its own distinct action set. Thus, the sets of available actions are purely local in the MDP formalism. In other words, each state could have its own private action labels. When modeling an *embodied* agent, though (which is the typical application of such models), the agent “takes its actions with it” (Polani, 2011), so here all or most of the actions transfer from state to state. It therefore makes sense to model them either as identical or at least similar especially when the effect is the same or at least similar across different states. We reiterate that this is in contrast to the core MDP which assumes neither the label sets to be the same across states, nor actions with the same labels across states having an analogous effect. In the following, we will make heavy use of this freedom to label action inconsistently.

#### 3.2.1 Semantics in the Action Labels

We will now discuss the issue of the action labels in more detail. One finds that action labels are often linked closely to the designed task. For a coffee-cooking task these labels would describe the interaction with certain ingredients or cookware, while in navigation each action label often corresponds to a specific global direction of the world the agent would move in, while in particular, this direction usually is set by the model designer to be coherent across states, such as selecting action *north* always moves the agent toward the global northwards direction. However, we emphasize that this is an assumption that, while sometimes true, cannot be generally made.

One finds in real life multiple examples for action labels which do not follow global properties and reflect local ones instead. Hiking paths for example are often presented to a hiker using signposts pointing to the local direction one is required to take to reach particular goals. In order to follow one particular hiking path on the mountain, hikers follow a provided identifier of the path, for example, a location name which identifies the action to follow the path independently from its relation to global directions. This way the information to follow this particular path is provided by the design of the action label (the path identifier). To reach the goal, this identifier is what the agent has to look for, and thus constitutes the information it needs to process.

Different agents can have different action labels while existing in the same world and having the same goal, making them look for different clues in the world. Two hikers could follow the exact same path, one using a globally oriented map and a compass to follow the global properties while the other uses the local signposts. The decision of whether to follow choose the next path based on the global direction or follow the signposts critically depends on the particular choice of embodiment. In a way, the coherent label arrangement in global navigation can be considered a well-adapted choice of labeling or an embodiment. It corresponds to a well-chosen link between action labels and the effect this has on the world dynamics or the task.

The agent’s choice of its internal labeling of its actions can thus contain information about the world or its task. As an external observer, we cannot make a definite assumption about the internal labeling. As an example, the action “going along a certain street” leading to the factory and the train station might have a different label for the factory worker (way to factory) and the commuter (way to station). In other words, a “natural” link between action label and the expected effect on the agent in its world may be present, but is always an *additional* assumption which we—in our model—aim to study separately. We will now construct a formalism that enables us to separate out the implicit assumptions made in typical MDPs, especially those embedded into a geometrical context, which is called “Twisted World”.

#### 3.2.2 Twisted World: Formalization

The *Twisted World* is a modification of a given MDP that aims to locally remap the action labels onto the transitions. During this process, we ensure that all actions lead to different transitions and all transitions remain feasible. Thus, in terms of action possibilities and under a traditional MDP perspective where each state *s* has its own—possibly singular—action set, the problem to solve the MDP remains precisely equivalent. However, it will turn out that this change has significant when we consider the informational cost of the resulting policies, as these look substantially different after the remapping (Polani, 2011).

We now formalize the concept of twisted world and follow the formalism of (Polani, 2011). Let $\sigma_{s}$, a permutation A→A, on the action set be defined for all states *s* of the MDP $\left(P_{s,a}^{s^{\prime }},R_{s,a}^{s^{\prime }}\right)$; $\sigma_{s}$ is in general different for different states *s*. Then, define the *twisted MDP* $\left({\tilde{P}}_{s,a}^{s^{\prime }},{\tilde{R}}_{s,a}^{s^{\prime }}\right)$, by ${\tilde{P}}_{s,a}^{s^{\prime }}=P_{s,\sigma_{s}\left(a\right)}^{s^{\prime }}$ and ${\tilde{R}}_{s,a}^{s^{\prime }}=R_{s,\sigma_{s}\left(a\right)}^{s^{\prime }}$. Note that this keeps the action edges in the state transition graph untouched and remaps only the action labels.

The relabeling affects any actions taken; in the case of action scripts the function σ is applied to every basic action *a* in the script: $\left(\sigma_{s_{1}}\left(a_{1}\right),\sigma_{s_{2}}\left(a_{2}\right)\ldots \sigma_{s_{n}}\left(a_{n}\right)\right)$ where $s_{i}$ is the state of the agent before the *i*th step of the script is carried out and action $a_{i}$ is about to be taken to obtain its effect on the twisted scenario. In particular, the length of the scripts remains unchanged.

Note that twisting does not change the optimal value for a given state *s*: $\tilde{V}\left(s\right)=V\left(s\right)$, and optimizing the MDP will find the same value. The effectively executed state transitions of optimized policies for both scenarios, twisted and untwisted, will be the same, but the policies, as seen by the agent’s perspective (who selects the actions by their labels) need to be remapped. This remapping affects the *Q*-function of the new MDP: since the policy $\tilde{\pi }\left(s,a\right)=\pi \left(s,\sigma_{s}\left(a\right)\right)$, the resulting *Q*-function becomes $\tilde{Q}\left(s,a\right)=Q\left(s,\sigma_{s}\left(a\right)\right)$.

The well-adaptedness of labels with respect to the embedding in the world is an implicit assumption on the structure of the world. In fact, it is a type of information that pervades the whole state space, as it implies a coherent orientation across states. The “twisted world” model permits us to limit or remove this implicit assumption. Beginning with a well-adapted action labeling, we will now consider twists σ of different degree. One consequence is that in these *twisted states* the action labels do no longer denote actions with a globally consistent effect, removing any assumption of action consistency throughout the world. We call all states s where $\sigma_{s}$ is not the identity *twisted states* and a world containing at least one twisted state a *twisted world*.

#### 3.2.3 Twistedness of a World

We now define the *twistedness* (*degree*) of a twisted world to be the ratio of the number of twisted states $\left(\left|s_{\text{twisted}}\right|\right)$ and the total number of states (|s|).

$$\text{Twistedness}=\frac{\left|s_{\text{twisted}}\right|}{\left|s\right|}.$$ (4)

The twistedness of the world shows how much of one state’s perception-action loop is transferable to the other states. An intuitive example for a partly twisted embodiment is how keyboard shortcuts change their meaning when a human switches from one software to another one. While *control + z* might mean *undo* in both programs, another shortcut might mean to different things, such as *control + d* meaning *delete* in program one while meaning *duplicate* in program two.

#### 3.2.4 The Perfect Twist

Twisting the world allows for an interesting anomaly: perfect twists. A perfectly twisted world is a world in which the agent can follow just one action label to reach the desired outcome we assume to be fixed. In a world in which all actions are linked to the global directions, the agent cannot move into different directions following just one action label. In a twisted world, this is possible. Carefully selected twists can create direct paths leading the agent to the desired outcome from any state using just one action label.

In randomly selected twists, however, it is, of course, highly unlikely that such a perfectly goal-adapted twist occurs. On the other hand, in nature, we observe many behaviors that can be derived by following a simple gradient or feature of a sensor stimulus such as optical flow, angle to a light source for flying insects or similar (Srinivasan, 1994). In this case, the sensor can be seen as implicitly activating the “same” action for as long as the stimulus exists. With nature setting the example, this technique has been adapted to be used in artificial unmanned aerial vehicles (Miller et al., 2019). Of course, the organism needs the ability to sense the gradient or a suitable proxy for it to generate such a label which obviously is easier for some tasks than for others. Also note that in general, such policies will be simple, and informationally cheap, but not be perfectly optimal in terms of traditional rewards.

In our examples, we limit ourselves to—in principle—fully observable worlds, so the sensor does not contribute to the simplicity of the action selection. In this sense, the perfect twists are the perfectly adapted embodiment, purely in terms of action labeling, to the world and the task.

### 3.3 The Cost of Decisions

Introducing scripts allows an agent to make a single decision and multiple actions. Hence, the agent can execute actions without deciding on it in every time step of the world. This allows us to pinpoint at which states in the world the agent takes a decision and where it does not. This further, allows us to model the cost of a decision only when one occurs. We consider two aspects when putting a “cost tag” on decisions, one is our informational costing of decision-making, and secondly, achieving a more refined analysis also permits the inclusion of an explicit cost for taking a decision at all. This will be a cost, expressed in value units which encompasses the sensory cost, information processing, and the process of initiating the decision itself. Note, this cost is separate from the informational treatment and we will model it as an abstract cost in the reward function. As in other mentioned work, the informational cost of the decision will, in contrast, be quantified in terms of Shannon information using the relevant information formalism (Polani et al., 2006).

#### 3.3.1 Definition of a Decision

To define the cost of a decision we first define “decision” in our context. Usually, people using the term “decision” mean the trigger point of a plan or an action after considering multiple possibilities (Endsley et al., 1997). The information to choose the action has to be actively searched for, then being processed and the action has to be triggered. However, in nature there many examples of actions triggered by pure stimulus response, such as the reflexes of a cat trying to balance itself (Hurteau et al., 2018) or even to flee (Willows and Hoyle, 1969). Those triggers are suspected to be directly linked in the neural controller from the sensor to the actuators. However also in those cases, the sensor is collecting information which is then processed and the result triggers an action. Thus, these action triggers not “free” in terms of cost. Both triggers require information processing and energy to keep the neural system running. We abstract the internal structure of the decision making and consider both cases a decision and model them as a stimulus response in the MDP framework.

#### 3.3.2 Relevant Information

An agent in the MDP framework needs to decide on an action based on the state it is in and thus needs to know in which state it is. Thus, the agent needs a certain amount of information about which state the current state is. Essentially, the cost is expressed here as the “per-step” relevant information that the agent needs to process about the current state to select the appropriate action which can be seen as the actual coding cost for the message sent from the sensor to the actuator (Tanaka and Sandberg, 2015; Tanaka et al., 2016). When an action (more precisely, action distribution, as the policies are permitted to be stochastic) is common among multiple states, these states do not need to be distinguished since this does not affect the decision. Not having to distinguish such two states reduces the information the agent needs for the decision. For instance, in an extreme case where the optimal action for the agent would be everywhere the same the information necessary to be processed for a decision vanishes. Where zero information cannot be achieved, our assumption is that the necessary information will still be minimized. Relevant information as a measurement was introduced in (Polani et al., 2006) and quantifies the minimal amount of information that needs to be processed during a decision given a certain (minimum) value constraint. The individual probabilities of all actions are defined by the policy. The mutual information between state and action is a quantity depending on the policy and the probability of being in a given state.

Formally, the *relevant information* of an MDP has been defined as the minimal amount of Shannon information required about the current state to select an action and achieve a given average utility E[Q(S,A)] (Polani et al., 2006). As the agent has no memory in this formalism, it has to acquire new information about its current state at every step. The relevant information is defined as the minimally achievable average of such information to be acquired across all states of the MDP, even though the specific amount of information necessary might differ from state to state. For the purposes of this paper, we will limit ourselves to optimal utilities only:

$$\underset{\pi \left(A|S\right)s.t.\;{\text{E}}^{\pi }\left[Q\left(S,A\right)\right]\overset{!}{=}Q^{*}\left(s,a\right)}{\min}I\left(S;A\right),$$ (5)

where I(S;A), the mutual information of the states and actions, is minimized in the policy and $Q^{*}\left(s,a\right)$ is the optimal value the agent can possibly achieve in a state *s*, taking action *a*. Therefore, relevant information finds the policy with the smallest mutual information among all policies achieving the optimal value.

This is a constrained optimization, which we solve by introducing Lagrangian factor β:

$$\underset{\pi }{\min}\left(I\left(S;A\right)-\text{βE}\left[Q^{*}\left(S,A\right)\right]\right),$$ (6)

β needs to tend to the infinite limit to achieve optimality of the value. The expectation *E* is taken over the joint distribution of all states *S* and all actions *A* given by p(s,a)=π(a|s)p(s). Since, I(S;A) is a concave function of p(x) for a fixed p(y|x) and a convex function of p(y|x) for a fixed p(x), the current minimization problem is similar to the rate-distortion problem (Berger, 2003) with a different fixed point. Hence, we use the same algorithm, the Blahut-Arimoto fixed-point iteration (Arimoto, 1972; Blahut, 1972) to solve the problem and to find the information-optimal policy which allows for the minimal channel capacity, as in traditional information theory, between sensors and actuators. Note that this means that, even for an—in principle—fully observable world, the sensor does only have to have this capacity and it needs to be tuned only to the relevant features to achieve optimal performance. It is important to note that the formalism only measures the amount of information, not the individual features. The minimized mutual information of state and action I(S;A) defined by (2) and obtained by the aforementioned Blahut-Arimoto algorithm is defined as the relevant information of the MDP solution and as such the minimal channel capacity necessary (Polani et al., 2006). If the agent does not have sufficient channel capacity from its perception of the world state to selecting its action, the optimal policy can not be reliably carried out.

Throughout this paper, we will assume a uniform distribution p(s) of the states for which the decisions are being taken for these calculations. Hence, every state is equally probable and actions occurring only in a small number of states or even only one contribute a lot to the mutual information. If these are not absolutely necessary, these actions will be removed during the minimization process. Thus, assuming the states to be uniformly distributed, the relevant information analyzes how uniform the actions across all states can be.

#### 3.3.3 Non-Informational Decision Cost

Knowing when decisions occur allows us to analyze them further. We do this by introducing a new component to the reward-function, which depends on the occurring decisions. Combining both rewards, we get a new reward function of:

$$r\left(s,a\right)=r_{\text{action}}\left(s,a\right)+r_{\text{decision}}\left(s,a\right).$$ (7)

This function allows to create a cost for a decision in addition to the cost of performing an action. We will use this reward function throughout the paper where, depending on the study, we will set $r_{\text{decision}}\left(s,a\right)$ to be zero (without decision cost) or one (with decision cost). The magnitude of the decision reward compared to that of carrying out an action is a model decision, and we currently do not have a principled way of choosing it. One could consider this cost as the actual energetic cost of actually running a decision as compared to the informational cost, which we propose to interpret as the allocation of limited, but pre-existing computational resources to take it.

## 4 Methodology

### 4.1 Scripts in Untwisted (“Well-Structured”) Worlds

After introducing scripts as an extension to the action space for RL we will investigate how this changes the agents’ behavior in a simple grid-world navigation task how the cost of decision factored into the reward function will affect this.

#### 4.1.1 The World

During the following experiments, we will consider a small grid world of 5×5 states, one start state, at least one goal state, and the set of basic actions A={north, east, south, west}. The MDP thus is defined on the full Cartesian product of states and actions, in other words, each state has the same selection of available action labels. The basic actions are interpreted as a movement toward the respective global direction indicated by the label unless stated otherwise. The agent carries no internal orientation and is thus always globally oriented (i.e. it does not keep to track of a direction it points to). A scenario with these properties will be henceforth called “well-structured”. Furthermore, we assume there exists a goal state or a set of multiple goal states with the properties defined in Section 2.2, effectively interrupting ongoing scripts. We model all transitions costs as rewards with the same value −1, and the cost of a script as the total reward accumulated during the running of the script (unless interrupted by reaching a goal state, see Section 3.1.3). The grid is finite and its outer borders consist of “walls” preventing the agent from leaving. An action causing the agent to walk into the wall leaves the agent’s state unchanged but still incurs the usual cost of 1. Since here we only consider optimal policies, no agent will waste effort walking into walls.

We will sometimes model an explicit decision cost. In this case, we define the cost of every single decision to be −1 (see Section 3.3.3). With this reward function, the agent has the incentive to make as few decisions as possible, while still taking the shortest path. This minimal model is sufficient to separate the effect of embodiment and of scripts to give insights into the effects of the internal encoding of the basic actions.

#### 4.1.2 Goal State Sets

**FIGURE 2:**
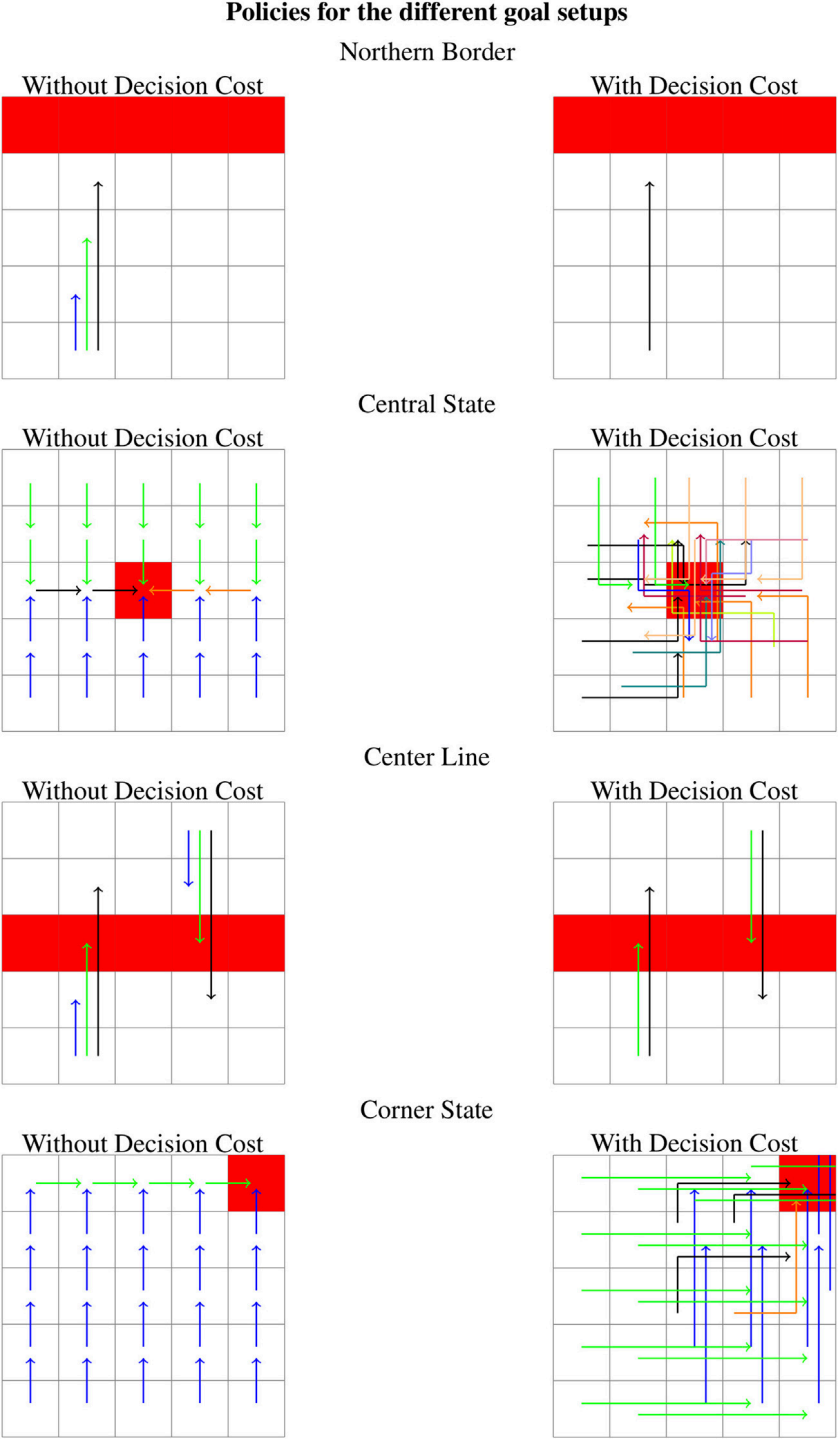
Resulting policies for the different goal sets with and without decision cost. Different colors denote different actions (basic actions or scripts). Thus all states with a differently colored action are the ones that need to be distinguished while the states with the same color can be interpreted by the agent as a single super-state which does not need to be further resolved.

We will look at four different goal setup, either single states or a set of states of varying informational hardness (for basic actions), which will permit exploring how different aspects of our (admittedly minimalistic) problem “morphology” changes affect the information balance of the problem. The individual goal state sets are shown in red in Figure 2.

Northern Border: This goal is composed of all northernmost states of the world. With only basic actions available, the relevant information is zero as the optimal action in all states is the same, going north.
Central State: Only the central state, the state furthest from any border of the grid, is a goal state. Reaching the goal requires different actions from different sectors on different quadrants of the goal. Thus the relevant information is comparably high with 1.28 bits.
 Center Line: Here a whole line running through the center is set as goals. With only basic actions, the policy contains only two of the four different basic actions driving the agent toward the goal with a relevant information of 0.80 bits.
 Corner State: Here, the goal is one state in one of the corners of the world. The policy for this task contains two actions and a relevant information of 0.25. As with the “Center Line”-setup, we only require two actions to reach the goal, but one of the actions is far more likely than the other, to achieve a lower mutual information.

### 4.2 Scripts in Twisted World

For the next experiments, we will remove the well-structured internal labeling of the actions by employing “twisted worlds” with varying degrees of twistedness (explained in Section 3.2). The general setup of the world stays the same: a grid world of 5×5 states with the Cartesian product of states and actions and the goal setups described in the last section. We here furthermore apply a twist σ to a subset of the states. We ensure that these twists, while randomly chosen, are selected ahead of any actual runs, and thus are fixed throughout an experimental series.

At the center of this experiment is the question of how the level of twistedness affects the information processing cost and the use of scripts for the given tasks. Keeping in mind that different twist at the same level of twistedness can be favorable to a task or not (see Section 3.2.4), we will average over multiple random twists with the same twistedness.

### 4.3 Single-Action Trajectories

We now explore a special type of policies: Zero-Information policies. In a way, these policies can be considered as providing a “fingerprint” of the dynamics of the world and, in case of a twisted world, the precise structure of the twists. Concretely, here, we will investigate how close to potential goals the actions (basic actions and scripts) lead under a Zero-Information policy. More precisely, instead of specifying a goal, and computing the cost of an optimal goal-directed policy at minimum information, we take the converse approach: we select a Zero-Information deterministic policy and establish how close to potential target destinations the agent can get following these in a twisted world. The idea of this experiment is to measure how effectively an agent can traverse a world using no information, i.e. sensory input, that is, in open-loop mode.

#### 4.3.1 Zero-Information Policy

Formally, a Zero-Information policy is a policy for which the mutual information is zero I(S;A)=0. Effectively the agent decides to do the same every time step. Though, this can be a probabilistic policy allowing the agent to choose between different actions, as long as these choices are equally probable at every point, for our analysis, we require a deterministic policy: using a single deterministic action in every given state. For each individual action (or script), there is exactly one deterministic Zero-Information policy. Furthermore, a script can contain multiple different basic actions within it, while still adhering to the above open-loop requirement effectively repeating the same sequence of basic actions over and over.

#### 4.3.2 Open-Loop Trajectories

While traversing the world in an open-loop manner given a fixed action, the agent follows a certain trajectory. We will characterize the coverage of the state space for these “skeleton” trajectories through the world using two different metrics for each potential goal state.

The coverage of the Zero-Information trajectories is measured in the following way: For each state *s* and possible goal *g*, we consider the trajectory generated by a deterministic Zero-Information policy and extract for each state on the trajectory how much the optimal cost ($V_{g}\left(s\right)$ *distance to a possible goal* in units of MDP cost) for each of these states is and also how far (*steps along the trajectory*) one has to move to minimize this cost. In other words, if the generated trajectory (assuming the movement is deterministic) consists of the states $\left(s_{0}=s,s_{1},s_{2},\ldots s_{k}\ldots \right)$, we define the two characteristics as $\min_{l}\left[-V_{g}\left(s_{l}\right)\right]$, the minimal distance (note that the distance is always the negative of the value in our scenario) from the open-loop trajectory at decision points to the given goal, and $\arg\min_{l}\left[-V_{g}\left(s_{l}\right)\right]$, how many steps the agent needed to take to get to the point of above minimum distance from its starting point $s_{0}$. Note that the agent can only leave a trajectory in states it “stops on” and takes a decision. Thus, all distances to possible goal states are measured only from these decision points which are not necessarily the best state to branch off from, (i.e. having the highest value of $V_{g}\left(s\right)$) on the trajectory. These two numbers are the metrics we investigate in the following. Smaller values indicate better coverage.

#### 4.3.3 Experimental Setup

For this experiment we will use a grid world as before but this time of the size 10×10. The (comparatively) larger world allows the agent to take multiple steps on the Zero-Information policy without reaching the end of the world. The coverage will be tested with all actions and different levels of twistedness. For the coverage experiment, the agent is not expected to reach a specific goal but simply traverse it according to its open-loop policy. Thus the world does not contain a goal. All states are evaluated based on their distance as per the above metrics.

## 5 Results

As general observation, the results show that, while scripts reduce the decision density, this only rarely does not incur additional informational costs while employing scripts usually either leads to a higher information cost in goal-directed tasks or leads to a loss in value when following open-loop trajectories. In twisted worlds, scripts are typically informationally more costly, but in rarer cases counteract the effect of the twists. We will start with the results comparing basic actions and scripts for goal-directed behaviors in well-structured worlds. Then, we proceed with the results in twisted world including a newly found phenomenon similar to the perfect twist which we will discuss below as “special twists”. Subsequently, we will present the coverage of worlds when using Zero-Information policies.

### 5.1 Scripts in Untwisted Worlds

For all experiments we only considered policies achieving the optimal value as defined in Section 2.2. Note that for some goal setups there can be multiple optimal solutions due to the grid nature and high level of symmetry of the graph. This is still the case after optimizing for relevant information.

Adding scripts to the action space without adding a separate decision cost does not change the relevant information for any of the goal sets as compared to only having basic actions available. The optimized policies for the “Northern Border” setup and the “Center Line” setup both contain scripts. The policies for the “Central State” setup and “Corner State” setup which constitute more localized goals only use basic actions (see Figure 2). Adding a decision cost to the reward function changes these results, as the optimal value is only achieved when the number of decisions is minimized, and thus the agent is forced to use scripts in every policy. For the “Northern Border” setup and the “Center Line” setup, the actions too short to reach the goal from any state are removed from the policy, but the relevant information does not change as it does not become necessary to distinguish between more groups of states than before. For the other two setups, the relevant information increases: from 1.28 to 2.14 bits for the “Central State” setup and from 0.25 to 0.66 bits for the “Corner State” setup. This means there are now more states that need to be distinguished from one another. In Figure 2, one can see that for the corner goal there without the decision cost two types of actions were used while the setup with decision cost uses four. For the “Central State” setup the agent needs to distinguish between even more states.

### 5.2 Scripts in Twisted Worlds

**FIGURE 3:**
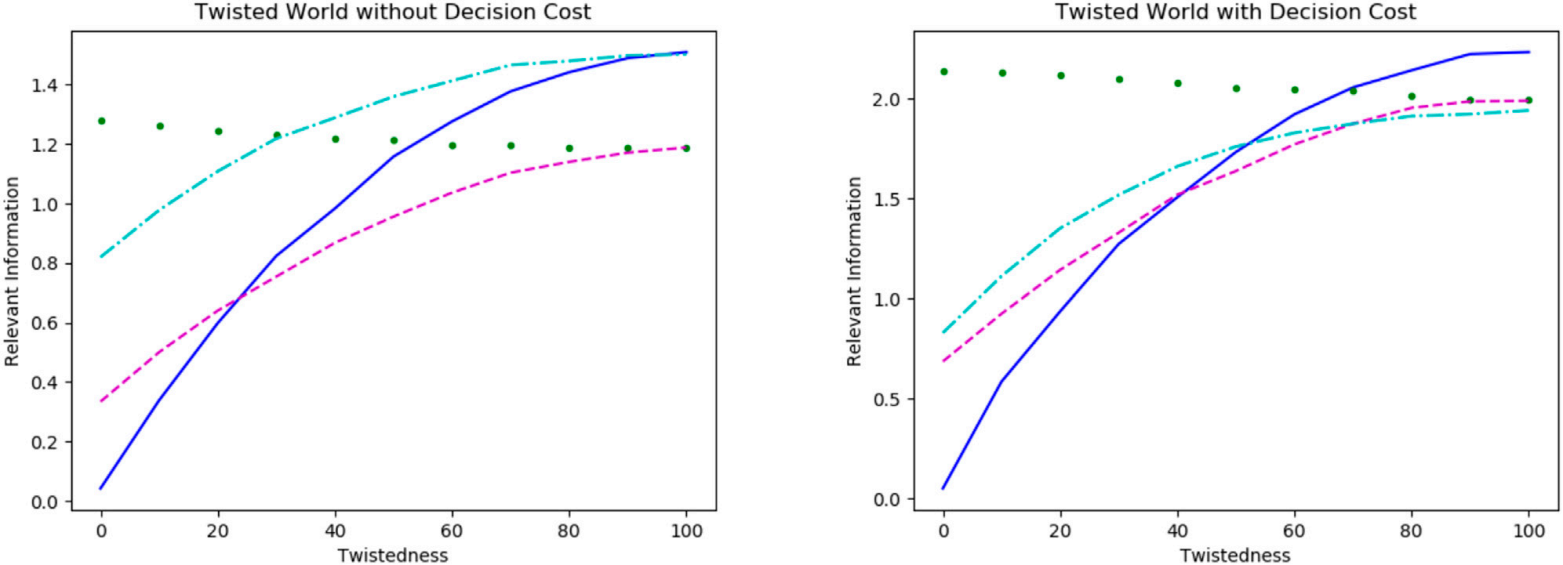
Relevant information depending on the twistedness of the world for the different goal setups: Border Goal in blue (line), Central State in green (dotted), Center Line in cyan (dash-dotted) and Corner State in magenta (dashed). Note, the scale for the value of relevant information is different in both plots.

For the experiments in twisted worlds, we considered the same world size and goal sets but every world contained a certain percentage of twisted states. We are interested in the relevant information and the occurrences of scripts in resulting policies, this time depending on the twistedness. Overall, the relevant information and the use of scripts becomes more similar across the goals with increasing twistedness of the world. The exact results are shown in Figure 3, left without and right with a decision cost and the colors indicating the different goal sets: Border Goal in blue (solid), Central State in green (dotted), Center Line in cyan (dash-dotted) and Corner State in magenta (dashed).

The relevant information rises for three (Northern Border, Central Line, Corner State) of the four goals and only goes down for the last one (Central State) which is not affected by adding a decision cost. This means that the untwisted world is the “easiest” world for three of our setups and the hardest for the goal in the center. In particular, the untwisted world proves to be unsuitably twisted for this particular goal.

Without a decision cost, scripts appear less often in the policies navigating the twisted world. Furthermore, the development of the relevant information for the “Central Line”-setup and the “Corner State”-setup is surprisingly similar. However the use of scripts is very different: The “Corner State”-setup uses scripts rarely, independently of the twists, while the “Central Line”-setup still uses scripts in the fully twisted world.

Interestingly, we see that the goals containing five states are equally difficult in the fully twisted world which is also the case for the two goal setups containing only one state. Thus, showing goals consisting of an equal number of states seem equally difficult to reach in a fully twisted world.

In the plot with decision costs, we see that all goals have a higher relevant information in a fully twisted world compared to those without it. Note, the scales of both plots are different. Since the agent is incentivized to minimize the number of decisions, every policy contains scripts. An interesting observation is that the “Northern Border”-setup (blue) which is not harmed by the decision cost in the untwisted world becomes the most difficult one in a fully twisted world. The “Center Line”-setup (cyan), initially not affected by the decision cost as well, shows a different behavior: It becomes the easiest setup in terms of relevant information on average at 100% twistedness.

Generally, the development of the setups with and without decision cost appears similar, but, on closer analysis, one finds that the decision cost has a big impact on the behavior in twisted worlds.

#### 5.2.1 Special Twists

Interestingly, there are “special” twists allowing the agent to use scripts without affecting the relevant information compared to the relevant information when only basic actions are available. These particular scripts fully negate the effect of the $\sigma_{s}\left(a\right)$, and thus, that of the twists, by encoding the relabeled action directly in the script.

**FIGURE 4:**
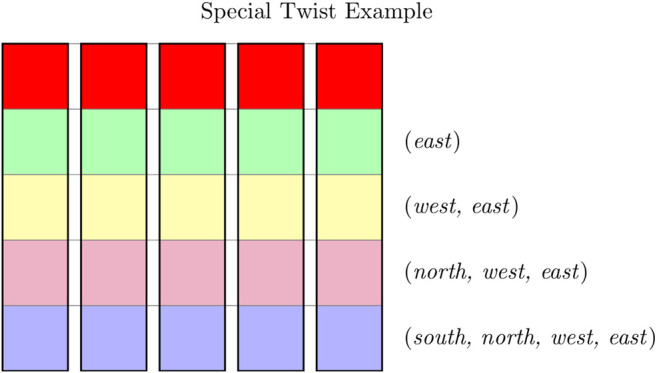
Example of a specific special twist for the “Northern Border” setup: All fields with the same color have the same twist regarding the action *north* (green: *east* → *north*, yellow: *west* → *north*, purple: *north* → and blue: *south* → *north*). Thus all five paths to the goal are the same and the resulting policy containing the scripts shown on the right side in information optimal.

An example of such a special twist for the “Northern Border” setup is shown in Figure 4. In this twist all states of each row have the same action label leading to the agent moving *north* (also indicated by the colors), concretely: green: σ(east)=north, yellow: σ(west)=north, purple: σ(north)=north and blue: σ(south)=north. Hence, when the agent takes a decision in any of the green (or any other single color) states it will experience the same twists on its path to the goal. Thus, this world is divisible into five different paths with the same regular occurrences of certain twists from the southernmost states to the goal. A script can model the future steps easily, in fact as easily as basic actions can. In this way, the scripts and basic actions next to the rows in Figure 4 show an information-optimized policy for this twist. Of course, the policy containing in each row only the first basic action is also an information-optimal policy but it is interesting that the scripts here are part of an information-optimal policy despite the high level of twistedness (at least 60%).

However, when those exact same twists do not occur all in the same row but are scattered in the world, the information-optimal policy only contains basic actions. It is important to note that this policy still has the same relevant information as with the ordered twists. Thus, we observe that scripts require more regularity to be used in an information-optimal fashion.

### 5.3 Single-Action Trajectories

The main objective of these experiments is to investigate the effect of twists on the coverage of the environment if a deterministic open-loop policy is run (either a single fixed basic action or a repeated application of a fixed script).

The effect of this depends on both the twist and the action (basic action or script) selected. In general, we find that the scripts get to the other states in fewer decisions and that the distances in twisted worlds tend to be higher. The detailed results are shown in the following subsections.

#### 5.3.1 Untwisted World

**FIGURE 5:**
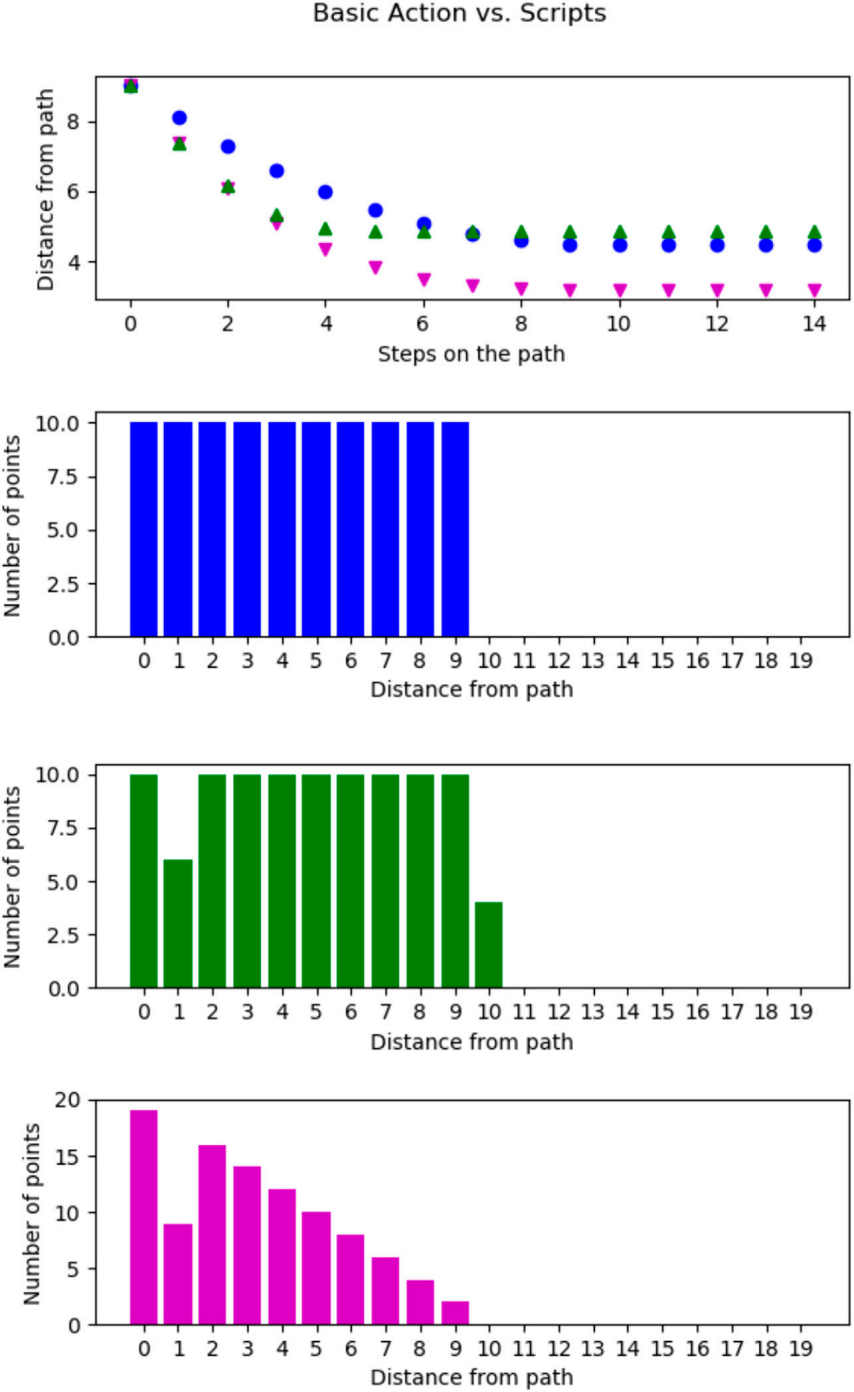
Comparison of the single action trajectory of the basic action (*west*) in blue (•), the script (*west, west*) in green (▲) and the script (*west, north*) in magenta (▼) from the southeast corner of the world. Top, the average distance ($V_{g}$) of the possible goal states, (e.g all states) per step on the trajectory. Second, number of states with a given distance $V_{g}$ from the agents movements for the basic action (*west*). Third, number of goals with a given distance $V_{g}$ from agents movement for the script (*west, west*). Fourth, number of goals with a given distance $V_{g}$ from agents movement for the script (*west, north*).

First, we compare a basic action to two different scripts shown in Figure 5. In all cases the agent started in one particular state, the southeast corner to make sure the setups are as similar as possible. Then the agent executed either the basic action (*west*), shown in blue (108), the script (*west, west*) shown in green (115), or the script (*west, north*) shown in magenta (116). Using any of these actions, the average distance to the possible goal states, (e.g all states) decreases when moving until the wall around the world is reached which stops further coverage. The basic action and the script (*west, west*) both move along the same trajectory. Both actions visit the ten states directly shown by distance zero as shown in the second and third plots of Figure 5, which shows the number of states at a given distance after reaching the wall. However, compared to the basic action, the script has fewer states with a distance of $V_{g}=1$ but some with a distance of $V_{g}=10$. This is due to the fact that the agent cannot branch off the trajectory in any state but only at decision points. Thus, we observe in the first plot that the distances per decision decrease more with every decision for the script but it does not reach the same distances overall because of the missing decision points. For every other state as the fixed start state, this looks similar, though the wall is often reached in fewer steps.

Now, we look at a script that actually includes different basic actions, a capability only possible after the introduction of scripts, here (*west, north*). In the last plot of Figure 5, we show the number of states at a certain distance for this script. This script visits 19 states directly ($V_{g}=0$) which is far more than the other two actions. Furthermore, this script also achieves $V_{g}=1$, $V_{g}=3$, and $V_{g}=4$ for more states than both other actions, and thus achieves a lower average distance shown in the first plot in magenta (▼).

**FIGURE 6:**
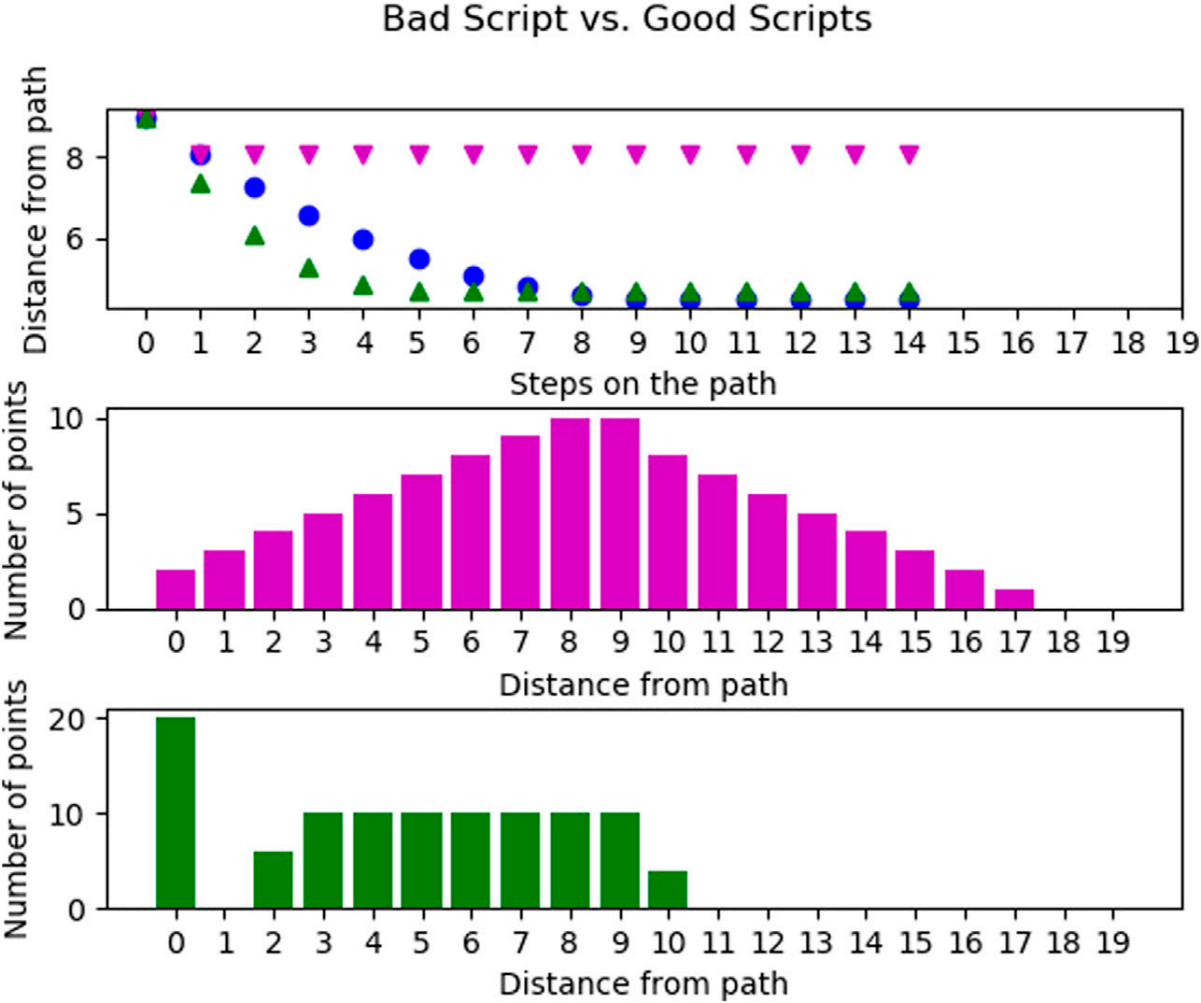
Comparison of the single action trajectory of the script (*west, east, west, east*) in magenta (▼) and the script (*west, north, east, north*) in green (▲) from the south east corner of the world. Top, the average distance $\left(V_{g}\right)$ of the possible goal states per step on the trajectory. Middle, number of goals with a given distance for the script (*west, east, west, east*). Bottom, number of goals with a given distance for the script (west, north, east, north).

Next, we compare two scripts we found to achieve a dissimilar coverage of the environment: In Figure 6, we compare two scripts (*west, east, west, east*), shown in magenta (▼), and (west, north, east, north), shown in green (▲). Again the agent started in the southeast corner. Further, the basic action (in blue (●)) from five is added as a baseline. The first graph shows that the magenta (▼) action does not get any closer to the other states after the first executed step while the green (▲) one does. In Figure 5, the script traversed the world faster than the basic action. However, this is not the case for the script (*west, east, west, east*), in fact, this scripts only ever visited two states. We will refer to scripts with this behavior as “non-traversing” in the future. The script (west, north, east, north) on the other hand achieves smaller distances to many states than the basic action. In fact, from the third plot, we see that 20 states are directly visited by this script.

Both of these scripts are extreme cases found during the experiments with all scripts. Thus, the other scripts fall somewhere in between those extremes. This shows, however, that the different scripts are of highly different usefulness for traversing the world.

**FIGURE 7:**
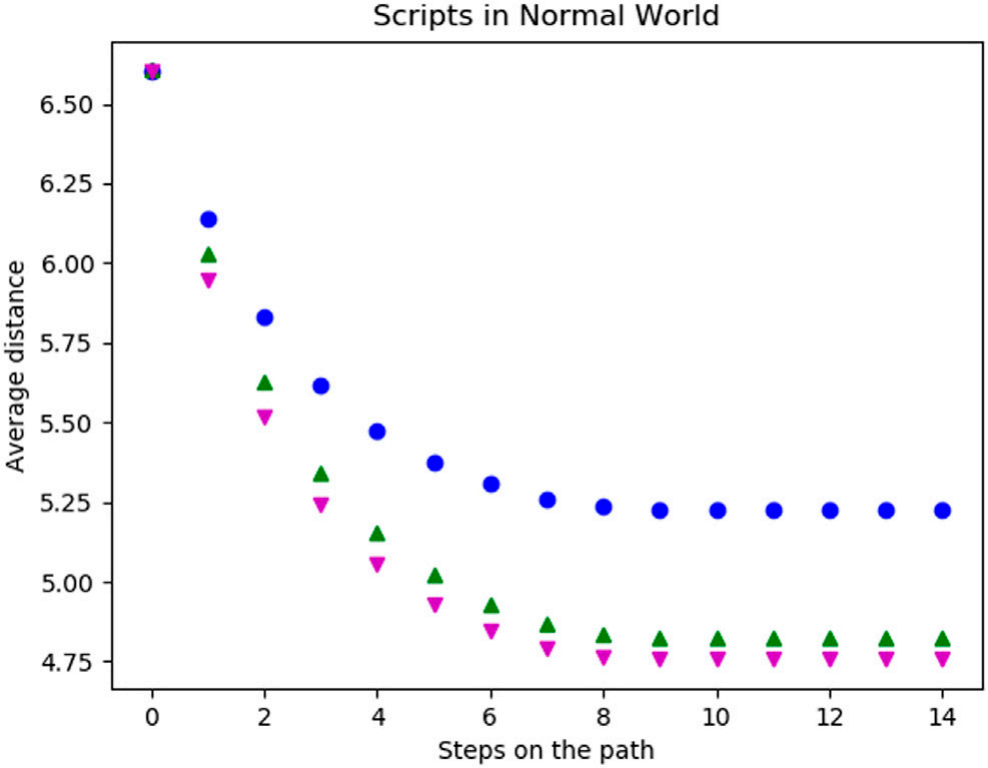
Average distance $\left(V_{g}\right)$ of the possible goal states per step on the trajectory for all scripts of length two in blue (●), all scripts of length three in magenta (▼) and the scripts of length two when removing all scripts only oscillating between two states in green (▲).

Lastly, we compared scripts of different lengths using the average distances per step averaged over all scripts of a certain length and all states of the world as starting states. The blue (●) line in Figure 7 shows the performance of all scripts with a length of two. Motivated by the results from the last paragraph, we removed all scripts with the worst performance (never visiting more than two states) from the script space. The resulting distances drop significantly and are shown in green (▲).

The magenta (▼) points refer to the average distances achieved with scripts of length three. With these the distances not only decrease in fewer steps but also decrease further compared to the scripts of length two. This is due to the fact that this action space includes very far moving actions such as (*west, west, west*) but also those which only effectively move one step such as (*north, south, west*). The second type of action traverses the world more slowly but actually improves the distances to some possible goals. Furthermore, there are no scripts of length three visiting only one or two states in all start states. Thus, we cannot prune low-performing scripts of length three as simply as with scripts of length two. All longer scripts behave similarly with the evenly long scripts including some non-traversing ones and the unevenly long scripts not.

#### 5.3.2 Twisted World

Twisting the world creates different paths the agent takes when it always repeats the same action. Thus, it can visit different states than before. Furthermore, the twists make it likely that the agent will visit a state multiple times after an initial transient. A typical trajectory is therefore composed of an initial transient and an “attractor” which in general will be a cycle, but can degenerate to a cycle of size one, that is, a fixed point. On the other hand, there always exists a perfect twist (see Section 3.2.4), in the sense that one can create a path visiting every state by always repeating the same action. As the agent has to visit all states, including the four corners which only have two possible basic actions allowing them to leave, the other two basic actions must leave the agent in the corner. Thus, there cannot be a twist creating “all-covering” Zero-Information trajectories for every action. Hence, when averaging over every available action and all states as starting state the perfect twist for one action is negated by the performance of the others.

**FIGURE 8:**
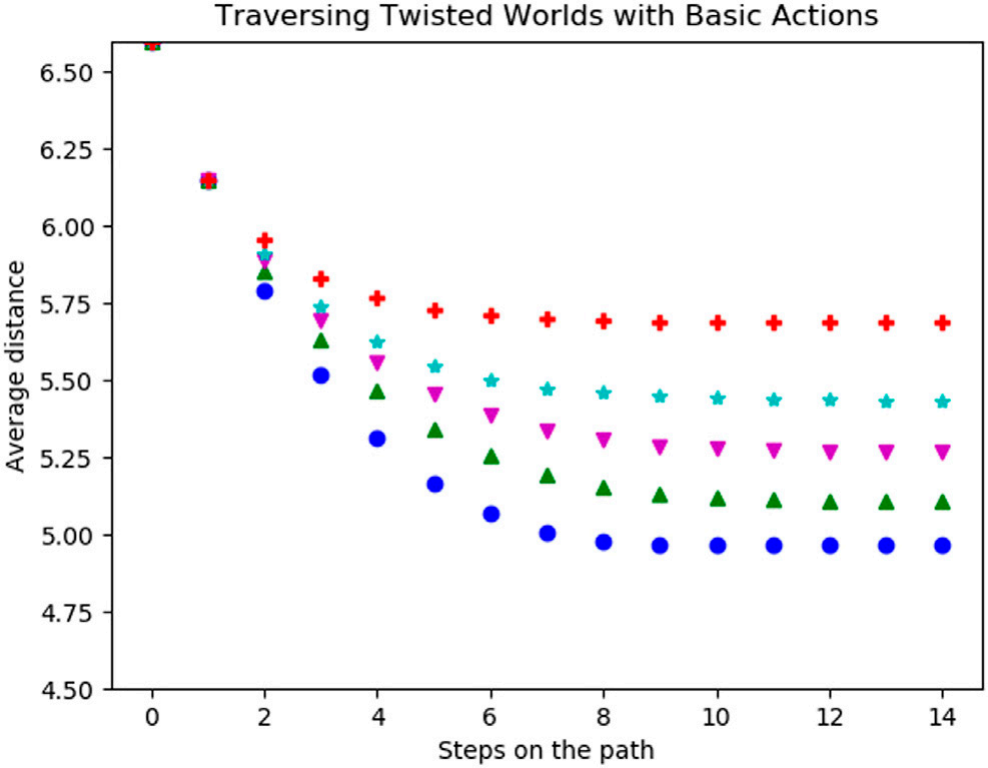
Average distance $\left(V_{g}\right)$ of the possible goal states per step on the trajectory for all basic actions in worlds with different twistedness: 0% in blue (●), 20% in green (▲), 30% in magenta (▼), 50% in cyan (★) and 100% in red (+).

During the first experiment, the agent was only able to use its original basic actions. In Figure 8 the average distances per step of five different levels of twistedness (0% (blue (108)), 20% (green (115)), 30% (magenta (116)), 50% (cyan (72)) and 100% (red (58))) are shown. For the starting state, all worlds have the same distances to possible goals. After the first step, the average distances are still the same. The reason for this is that the agent either moves to a new field or walked into a wall. However, the number of actions leading into a wall does not change because of a twist and thus the same amount of first steps move into the wall. In the following steps, the average distance decreases until the agent, independently of the start state, does not move any further. The coverage of the environment highly depends on the twistedness, as the agent traverses the fully twisted world the least and the untwisted world the most as the latter achieves the lowest values in our metrics. Worlds with intermediate twist ratios fall in between.

**FIGURE 9:**
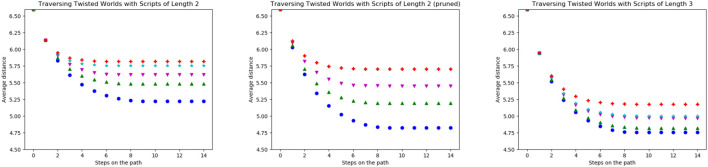
Average distance $\left(V_{g}\right)$ of the possible goal states per step on the trajectory for all scripts with length two **(left)**, pruned scripts with length two **(middle)** and scripts with length three **(right)** for worlds with different twistedness: 0% in blue (●), 20% in green (▲), 30% in magenta (▼), 50% in cyan (★) and 100% in red (+).

Traversing the twisted worlds with scripts (shown in Figure 9) shows a similar result to the basic actions: The more twisted the world is, the less the agent traverses the world. Interesting is that removing the previously bad scripts (see Figure 7) only achieves comparably little improvements in terms of the distances, especially for highly twisted worlds.

**FIGURE 10:**
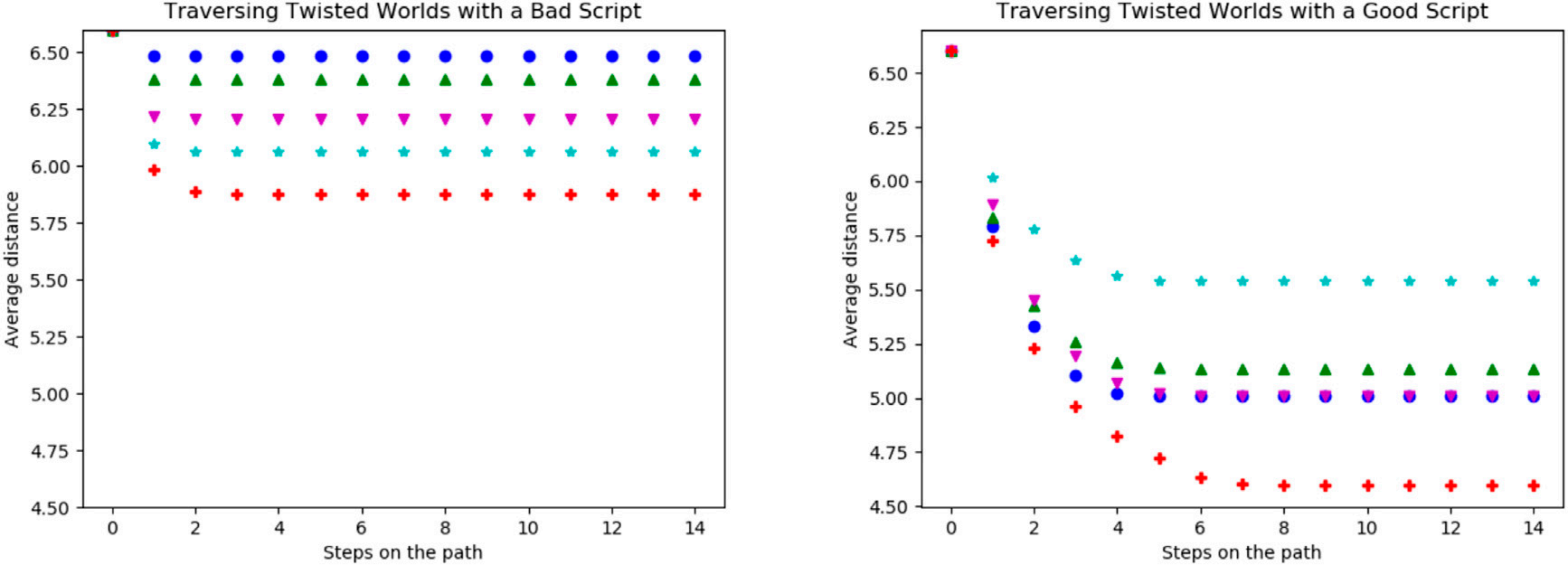
Comparison of the trajectory of the script (*west, east, west, east*) on the left and the script (*west, north, east, north*) in the right in worlds with different twistedness: 0% in blue (●), 20% in green (▲), 30% in magenta (▼), 50% in cyan (★) and 100% in red (+). The achieved distances of the bad script show it is getting better the more the world is twisted. Interesting is also that the good script works exceptionally well in the 100% twisted world. This is due to the fact that for this script many possible good twisted world exist.

The reason for this is shown in Figure 10 on the left. Here we again compare the scripts (west, east, west, east) and (west, north, east, north) from Figure 6. There the bad script ((*west, east, west, east*)) is evaluated and actually becomes more viable with more states being twisted. It did not reach more coverage than the previously good script ((*west, north, east, north*)) in Figure 10 but far more than in the untwisted world. The previously good script, in the right in Figure 10, is affected very differently by individual twists and thus sometimes getting worse with twists but other times better with the twists. Since the red (58) curve is very low, we suspect that here many almost perfect twists occur for a fully twisted world (see Figure 10).

The achieved distances of the bad script show it is getting better the more the world is twisted. Interesting is also that the good script works exceptionally well in the 100% twisted world. This is due to the fact that for this script many possible good twisted world exist.

To summarize all results, twisting the world leads to less coverage of the world independently of whether basic actions or scripts are used. Furthermore, in the twisted world, it is less easy to clearly separate between “bad scripts” and “good scripts” because the twist can turn originally advantageous scripts into ineffective ones and vice versa.

## 6 Discussion

This paper contains three major points. First, the formalism for the creation of scripts from basic abilities to reduce the decision density; second, whether there exist scenarios in which scripts incur no additional control complexity for goal-directed navigation in both untwisted and twisted worlds; third, the merits of scripts for traversing the state space in general again comparing untwisted and twisted worlds. In all cases, we explored if and how scripts save resources for the agent and how the internal coding, the embodiment, of the basic actions impacts the cognitive cost.

### 6.1 Scripts

The core concept of a script is combining different basic actions to an action sequence accessible as a whole. Thus, the script is a composite action built out of basic capabilities to a new more complex capability. In reinforcement learning, the option framework comes to mind as achieving something similar: combining multiple actions to an option (Sutton et al., 1999). Both approaches (scripts and options) create new actions lasting over multiple time steps but are triggered only once and then executed. However, the execution of these actions is fundamentally different: While an option is a sub-policy, constantly processing information, and reaching certain termination criteria, a script does not take in any feedback from the world and terminate after executing all actions. Thus, the goal of both concepts is different: An option reaches a subgoal; a script executes pre-defined actions in a pre-defined order.

Scripts are modeled after fixed action patterns which are highly specific to the animal (Schleidt, 1974). Many of these patterns have their individual neural sub-controller, often found in the spinal cord of animals (Desrochers et al., 2019). Similarly, we assume the scripts themselves to be generated by a different control unit to which the agent can delegate the detailed execution (see Section 3.1.2), but which does not require deliberative resources. In this case, we consider the agent to be able to “outsource” this computation from its main decision making unit to the sub-controller of the scripts. How exactly to include external processing in the consideration of information cost is not discussed in the present paper and will be investigated in future work.

### 6.2 Goal-Directed Behavior

In an untwisted world, adding scripts to the action space has shown to be informationally optimal for some setups but not for others. In Section 5.1 the “Northern Border”-setup and the “Center Line”-setup have both shown to make use of scripts without a rise in relevant information. Thus, the agent can save decisions and does not need to process more information allowing one to save resources. For other goal setups chosen (“Central State” and “Corner State”), however, scripts are not selected after the optimization. So, the usefulness of scripts depends on the setup, that is, the task, even for a minimalistic one like this. Both setups (“Northern Border”, “Center Line”) using scripts result in a relevant information below 1 bit. This leads to the hypothesis that a low relevant information leads to the use of scripts. However, the “Corner State”-setup which does not use scripts has a low value of relevant information as well. Thus, the value of relevant information does not directly indicate whether scripts are useful or not.

For some setups, forcing the use of scripts by adding the decision cost to the reward increases the relevant information. In other words, the agent needs more precise knowledge (information processing) to take the decision what to do in a future step before it actually occurs, as seen in the results in Table 1 with decision cost for the “Central State”-setup and the “Corner State”-setup. This suggests that in some of these cases, taking a decision late on the path and increasing the number of decisions might be informationally cheaper in total, though this needs further investigation in the future. The difference of relevant information with and without the decision cost gives us an idea of whether scripts can be easily used for a particular setup. However, what causes the different goal setups to be script-friendly or not will be part of our future work.

**TABLE 1:** Relevant information and occurrences of scripts for the different goal setups.

	Without Decision Cost		With Decision Cost	
Setup	Relevant information In bits	Scripts used?	Relevant information In bits	Scripts used?
Northern Border	0	Yes	0	Yes
Central State	1.28	No	2.14	Yes
Center Line	0.80	Yes	0.80	Yes
Corner State	0.25	No	0.66	Yes

With the introduction of the twisted world, we removed the well-ordered labeling of the basic actions and thus any specific external design regarding the information about global directions, pervading the whole world. The twists decouple the encoding of triggering actions (in our interpretation of the concept of “embodiment”) and their effect on the world and thus, removes the morphological computation aspect of the environment. We now consider how these automatic open-loop scripts interact with the environmental twists and under which circumstances a good twist can reduce the agent’s cognitive cost.

In general, the results in Section 5.2 have shown the agent is required to control its actions more precisely and needs to process more information. For most of the tasks, the informational cost of the policies increases independently of the availability of scripts, the more the world is twisted. However, for the “Central State”-setup, the twists made the policies easier in terms of information. In fact, the “well-labeled” world required the most information, suggesting that the obvious (“trivial”) label is not necessarily the best for all cases. The action following a specific basic action is less predictable in a twisted world and thus, scripts are less used in twisted worlds even when available. When forced through a decision cost, the relevant information increases. A high value of the relevant information indicates that scripts are not easy to use for a particular task.

Even though most twists make scripts difficult, some twists allow for the use of scripts. When the twists occur with a certain regularity, the scripts are actually able to represent twists within itself. Hence, scripts can embody this regular irregularity created by a particular twist along all paths to the goal (see Figure 4). Furthermore, all states from which this particular irregularity begins can be grouped, as they are in Figure 4, with different lighter colors. In fact, the informationally optimized policy directly creates these groups of states with the assigned actions. Additionally, the scripts require the twists to appear with a certain regularity along the paths but basic actions do not require this. Thus, we hypothesize that scripts need more regularity in the world to be information-optimal than the basic actions. Hence, a regular embodiment is even more important when we want to reduce the decision density.

From these results for goal-directed behavior in twisted worlds in Section 5.2, we definitely see the importance of well-labeled actions, that is what we interpreted as “embodiment”, being adapted to the world and the goals. In cases when the label of an action corresponded to the same direction in every state, the actions are linked to a global feature of the world. We interpret this as the embodiment of the agent representing a semantic global feature of the world. In Figure 3 we see that breaking this embodiment more and more results in higher information processing for three of the setups. However, we also see this is not the case for the “Central State”-setup. Thus, the agent needs to process less information for multiple goal setups in an untwisted world but not for all.

Note here that the setups previously using scripts were not using the scripts anymore when the world was changed to a twisted one. Hence, to use scripts in an information-parsimonious context and thus, take fewer decisions, the embodiment of the agent and particularly its basic actions are important.

### 6.3 Traversing the World

In contrast to the previously discussed results, here the question was whether an agent reached a goal or how closely it could approach it without any information processing whatsoever. Next, we will discuss the affect of scripts and twists on the agent’s capability to traverse and cover the state space with limited information processing.

We observe a difference between an agent with scripts and one with basic actions: The first was able to traverse the world with fewer decisions. However, the average distance to the states which were not visited in this case was larger for the script repeating the same basic action. The reason for this is that the agent cannot branch off at any state of the trajectory but only when a decision is made. Thus, scripts allow the agent to move with fewer decisions but are in the above sense, less accurate. The other script (*west, north*) (see Figure 5) shows the potential of scripts containing different basic actions, visiting more states directly and achieving lower distances overall.

During all experiments with a twisted world, the agent came close to fewer states than in an untwisted world. Hence, twists of the labeling of the basic action make traversing and covering the world harder, independently from the availability of scripts. In twisted worlds, the agent ended up visiting the same states over and over, creating the projected trajectories composed of an initial transient and an “attractor” the agent never leaves. Thus, we can say that a well-chosen labeling of basic actions plays a major role in the coverage of a state space with Zero-Information policies.

This task cannot only evaluate an agent as a whole but also its individual actions, in other world evaluate a script. Using consistent labeling, scripts not traversing the state space are easier to spot. Since these scripts provide little value for navigation (only reaching two states in however many steps), these scripts can easily be identified as bad choices for the task, and thus adding a new sub-controller for it is a useless effort. Its potential relevance for modeling biological control mechanisms is that it is very important to know what not to spend resources on or into which skills not to invest. Hence, the information that a certain script is useless ahead of time is valuable information. However, in a twisted world, this decision is much harder. Almost every script has at least some merit somewhere in the world. In fact, the more the world is twisted, the more “traversing” all previously non-traversing scripts became. Hence, in an untwisted world non-traversing scripts are far easier to identify than in a twisted world.

## 7 Conclusion

We studied relevant information in the framework of reinforcement learning and action scripts as well as untwisted (well-labeled) and twisted (ill-labeled) worlds. We were especially interested in the effect combining actions to scripts has on the relevant information. We further studied the importance of the embodiment of the basic action labels by comparing the untwisted world to varyingly twisted worlds.

We defined a script, a fixed sequence of basic actions, as an extension to the reinforcement learning framework. A script creates new skills from the existing ones to composite actions, does not take feedback into account, is open-loop, and is similar to a fixed action pattern. Adding these to an agent, it now can decide on multiple future actions at once. However, adding all possible scripts creates a huge action space, and which scripts should be added to the action space, is an open question.

We have analyzed the changes in information processing when using the new action space in goal-directed tasks We clearly see that there are cases for which the introduction of scripts allows the agent to save decisions without processing more information for an average decision. For other cases, we see this is not possible. This suggests that, depending on the task, scripts can save decisions at no additional cost.

We then tested the scripts against a label-wise twisted world, that is, what we interpret in the abstract transition graph model of our world, as a different basic embodiment. Independently from the use of scripts or basic actions, the agent had to process far more information per decision. In most cases, the scripts would be used as little as possible. Overall, a high value of relevant information, whether because of the selected goal or the twistedness, makes the use of scripts less attractive. Interestingly, scripts have shown to work well with some special “ordered” twists in Section 5.2.1. This leads us to the conjecture that scripts need to exploit some sort of ordered feature of the world to be effective. In these cases, the script embodies the irregularity of the world.

Limiting the agent to a Zero-Information policy while traversing the world, we clearly see scripts needing fewer decisions than basic actions. Still, trajectories generated by scripts are less accurate compared to basic actions. A twisted world is generally badly covered, independently of the use of scripts. This shows that a consistent labeling of basic actions which provides a kind of orientational semantics to the embodiment is important to save information processing during the tasks. This technique further allows us to evaluate an action with respect to its use for traversing the state space. Non-traversing scripts are easily spotted when the world is well-structured, a step toward reducing the action space to good scripts. In a twisted world, however, these “bad actions” are no longer as obviously bad and therefore harder to spot. Thus, a consistent labeling allows to identify more easily which scripts are useless to have in the repertoire. A well-adapted labeling of the basic actions allows one to make decisions more easily (informationally cheaply). This is further enhanced by the ability to create scripts which reduce the number of decisions overall.

Among other, the results show that scripts are in general more sensitive to twists than basic actions. Why this is the case and what exactly causes it is a question for the future. We suspect that understanding this phenomenon will also give further insight into the cause when special twists as observed here occur which are then exploitable by scripts. In general, we see only some scripts being used at all. Thus, one goal of future research will be to predict such useful scripts (especially if they apply to more than one goal), and only adding these to the action space and thereby reducing the huge action space that the current method requires.

## Data Availability

All datasets generated for this study are included in the article/supplementary files.
